# Alacrymie congénitale révélant un syndrome d'Allgrove: à propos de trois cas

**DOI:** 10.11604/pamj.2015.20.359.4717

**Published:** 2015-04-14

**Authors:** Rajae Derrar, Nourredinne Boutimzine, Amina Laghmari, Amal Alouane, Rajae Daoudi

**Affiliations:** 1 Université Mohammed V Souissi, Service d’‘Ophtalmologie A Hôpital des Spécialités CHU Rabat, Maroc

**Keywords:** Alacrymie congénitale, population pédiatrique, Achalasie œsophagienne, Congenital alacrima, pediatric population, oesophageal achalasia

## Abstract

Le syndrome d'Allgrove ou triple A syndrome est une affection autosomique récessive constatée chez la population pédiatrique, associant dans sa forme complète: Achalasie œsophagienne, Alacrymie, maladie d'Addison (insuffisance surrénale), une dégénérescence neurologique et occasionnellement une instabilité du système autonome. Nous rapportons les cas de 3 enfants issus de mariages consanguins, chez qui l'examen ophtalmologique a révélé une sécheresse sévère avec dans deux cas une kératite envahissant l'axe visuel, ainsi qu'une paresse du reflexe photomoteur. Le bilan radiologique: transit œsogastroduodénal (TOGD) et fibroscopie œsogastroduodénale (FOGD) a révélé un mégaoesophage associé dans un cas à une œsophagite. Un traitement à base de larmes artificielles est instauré aussitôt, ainsi qu'un traitement chirurgical par voie laparoscopique. La connaissance de cette pathologie permettra une prise de conscience de la gravité de cette maladie en plus de suggérer sa prise en charge.

## Introduction

Le syndrome d'Allgrove ou syndrome des 3 A est une affection génétique de transmission autosomique récessive associant dans sa forme complète: Achalasie œsophagienne, Alacrymie et insuffisance surrénale. C'est une affection rare, moins de 80 cas ont été rapportés dans la littérature [1].

## Méthodes

Nous rapportons trois cas récents révélés par une alacrymie.

### Observation 1

**Figure 1:**
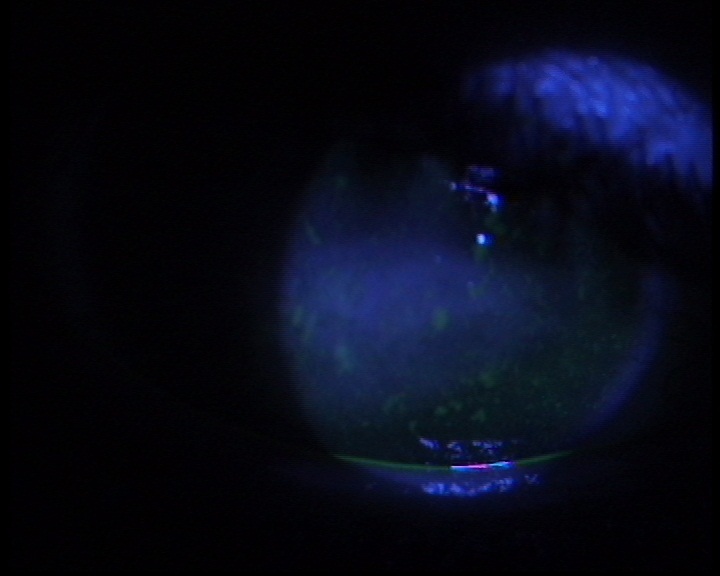
Aspect de kératopathie sèche chez un enfant de 4 ans révélé par un test à la fluorescéine

**Figure 2:**
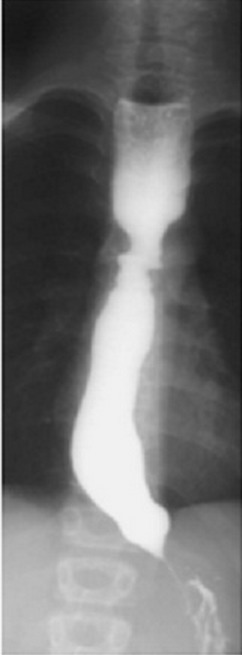
Transit œsogastroduodénal montrant un rétrécissement régulier du bas œsophage

Garçon âgé de 4 ans, 2^ème^ d'une fratrie de 5, issu d'un mariage consanguin consulte pour une baisse d'acuité visuelle bilatérale chiffrée à 2/10^ème^ au niveau de l’œil droit et à 3/10^ème^ faible au niveau de l’œil gauche non améliorable. L'examen à la LAF trouve une kératopathie sèche (Figure 1) avec un test de Shirmer significativement altéré. L'examen du fond d’œil est inappréciable. L'interrogatoire révèle par ailleurs l'absence de larmes même lors des crises de pleurs, une dysphagie paradoxale qui s'est aggravée avec un retard staturo-pondéral. La réalisation d'un transit œsogastroduodénal combiné à une fibroscopie œsogastroduodénale a montré un rétrécissement régulier du bas œsophage, facilement franchissable sans reflux ni signe d’œsophagite (Figure 2). Le bilan surrénalien est normal.

### Observation 2

**Figure 3:**
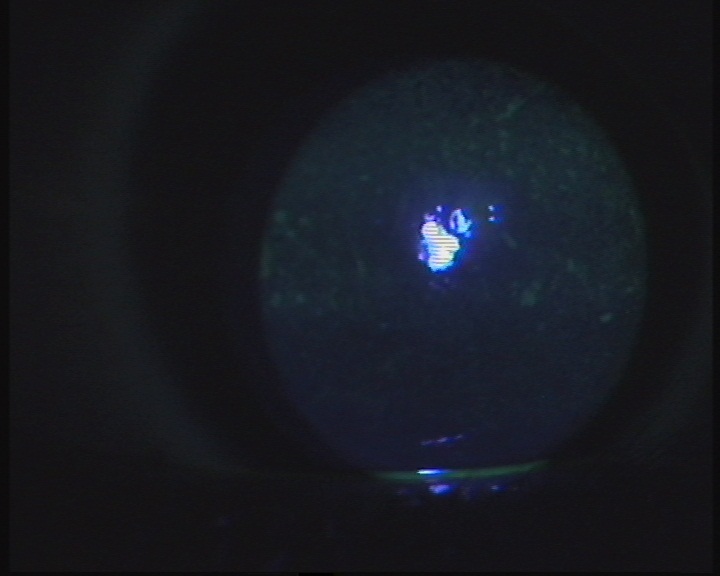
Kératite ponctuée superficielle au niveau de l’œil droit chez une enfant de 7 ans

**Figure 4:**
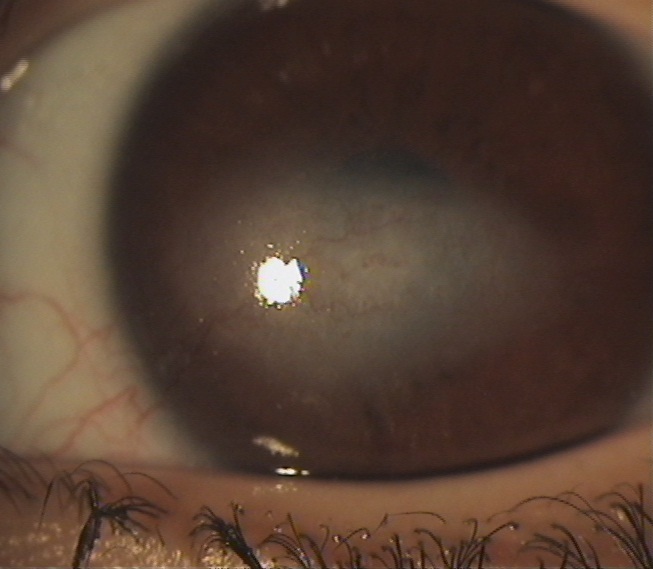
Taie de cornée centrale de l’œil gauche avec néovascularisation

**Figure 5:**
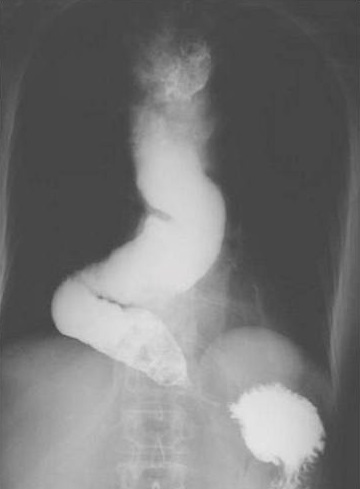
Transit œsogastroduodénal en faveur d'un méga œsophage

Fille âgée de 7 ans, 3^ème^ d'une fratrie de 4, issue d'un mariage consanguin présente une baisse d'acuité visuelle bilatérale chiffrée à 2/10^ème^ non améliorable au niveau de l’œil droit et à mouvement des doigts au niveau de l’œil gauche. L'examen au biomicroscope révèle une kératite ponctuée superficielle au niveau de l’œil droit (Figure 3) et une taie de cornée centrale de l’œil gauche avec néovascularisation (Figure 4). L'examen du fond de l’œil est normal à droite. Par ailleurs, ses parents rapportent la notion de vomissements alimentaires puis des épisodes de dysphagie paradoxale. L'examen biologique trouve des taux normaux de cortisol (453μm/l) et d'ACTH (27pg/ml). Le transit œsogastroduodénal est en faveur d'un mégaœsophage (Figure 5) et la fibroscopie œsogastroduodénale en faveur d'une œsophagite stade 1.

### Observation 3

**Figure 6:**
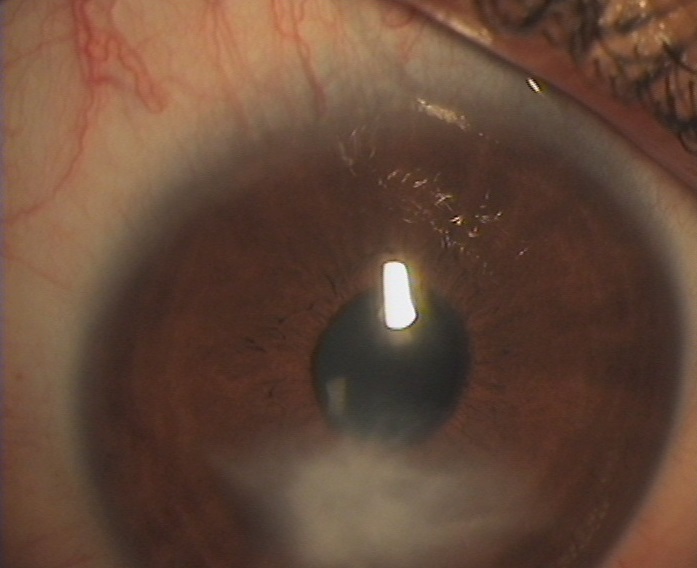
Taie de cornée de l’œil gauche chez une enfant de 5 ans

Fille âgée de 5 ans, 6^ème^ d'une fratrie de six, issue d'un mariage consanguin chez qui les parents ont constaté l'apparition d'une taie cornéenne de l'OG (Figure 6) et une Alacrymie bilatérale. L'acuité visuelle est estimée à 2/10^ème^ au niveau de l’œil droit, et à mouvement des doigts au niveau de l’œil gauche. L'examen au biomicroscope met en évidence une kératite bilatérale, plus importante à gauche, prenant la fluorescéine et un fond d’œil d'aspect normal. L'interrogatoire révèle la présence de vomissements et une dysphagie paradoxale avec un retard staturopondéral. Le bilan biologique est normal et le TOGD combiné à une FOGD met en évidence un mégaoesophage sans signe d’œsophagite.

## Résultats

Le premier patient a bénéficié d'un traitement à base de larmes artificielles à instillations fréquentes et régulières et l'achalasie de l’œsophage a été traitée chirurgicalement par voie cœlioscopique: cardiomyotomie de Heller associée à un geste anti-reflux avec de simples suites opératoires. Le deuxième enfant a bénéficié d'un traitement à base de larmes artificielles en attendant la greffe de cornée, et d'un geste chirurgical identique au précédent avec des suites simples. Le troisième enfant a bénéficié d'un traitement chirurgical du mégaoesophage par voie cœ'lioscopique avec suppléance lacrymale en insistant sur la nécessité d'une instillation continue. Le recul au delà de 6 mois en moyenne est marqué par la persistance de la KPS malgré un traitement assidu et l'absence d'amélioration de l'acuité visuelle. Sur le plan général, les signes digestifs ont nettement régressé en post opératoire avec une légère amélioration de la courbe de croissance staturo-pondérale. L'insuffisance cortico-surrénalienne n'est, quand à elle, pas apparue jusqu’à ce jour.

## Discussion

Le syndrome d'Allgrove est une affection rare (97 cas publiés), de transmission autosomique récessive, Le gène responsable de la maladie, localisé sur le chromosome 12, coderait la protéine ALADIN (pour alacrima-achalasia-adrenal insufficiency neurologic disorder), qui appartient à la famille des protéines de régulation. Le rôle exact de cette protéine dans le syndrome Triple A n'est pas encore connu. Mais les chercheurs pensent qu'elle pourrait avoir un rôle de régulateur sur les récepteurs de l'hormone produite par les glandes surrénales, le cortisol, et un rôle dégénératif sur le système nerveux [1, 2].

En 1978, Allgrove et all rapportent pour la première fois le cas de deux frères et deux sœurs, issus d'un mariage consanguin qui présentent une achalasie, une déficience de l'axe glucocorticoïde, et un défaut de sécrétion lacrymale, leur permettant de définir cette combinaison comme étant un trouble héréditaire familial connu sous le nom du syndrome d'Allgrove ou syndrome des 3 A [3, 4]. Dans les années suivantes, un certain nombre d'auteurs ont publié des rapports similaires qui ont contribué à définir les principaux caractéristiques de ce syndrome [3, 4].

L'incidence de la pathologie est inconnue et difficile à déterminer à cause de la variante clinique de la maladie et de la mortalité infantile due aux crises d'insuffisance surrénale, d'où l'importance d'une anamnèse soigneuse à la recherche d'antécédents de mortalité dans la fratrie. L'Allgrove touche aussi bien le sexe masculin que le sexe féminin sans différence de race (sexe rationnel= 1). L’âge de début est imprécis; l'achalasie et l'alacrymie représentent les signes inaugurateurs de la pathologie et peuvent apparaitre dès la naissance alors que l'insuffisance surrénale se développe au cours des deux premières décades de la vie [5]. Par ailleurs, d'autres auteurs ont suggéré le nom du syndrome des 4A: insuffisance surrénale, achalasie, alacrymie, anomalies du système autonome, mais tous ont conclu que l'alacrymie représente le signe inaugural de l'affection, suivi de l'achalasie [4–7]. Une association entre le syndrome de triple A et une neutropénie chronique asymptomatique a également été rapporté [3].

Les principales manifestations ophtalmologiques dans le syndrome triple A incluent l'alacrymie avec l'atrophie des glandes lacrymales et absence de larmoiement, les kératoconjontivites, des anomalies pupillaires notamment une lenteur du jeu pupillaire, des troubles de l'accommodation, une amblyopie et une atrophie optique [2, 8].

Les processus d'accommodation, de myosis et de larmoiement (basal et reflexe) sont sous contrôle parasympathique et cholinergique, ce qui explique que les troubles observés chez les patients souffrant du syndrome triple A résultent d'un dysfonctionnement du système autonome probablement en rapport avec une atteinte centrale [5, 7, 9–11]. Mullaney et al. [8] ont noté la présence de glandes lacrymales atrophiées et une réduction des glandes séreuses sur pièce de biopsie des glandes lacrymales chez trois de leurs patients. Les crises d'insuffisance surrénale peuvent être découvertes à l’âge adulte, lors de la 2ème décade, ou peuvent ne jamais apparaître [4, 5], et c'est le cas de nos trois patients. Cette insuffisance peut compromettre le pronostic vital et doit être recherchée soigneusement.

D'autres symptômes ont été constatés au cours de la maladie incluant: o Microcéphalie en rapport avec la récurrence des crises d'hypoglycémie et/ou la malnutrition, parfois une dégénérescence neurologique avec retard mental. o Hyperpigmentation, hyperkératose, fissuration de la paume des mains et de la plante des pieds. o Hypotension orthostatique en rapport avec la défaillance du système nerveux autonome. o Bronchopneumopathies à répétition due à la dysphagie et l'achalasie.

Le traitement de la maladie d'Allgrove comporte 3 volets: l'alacrymie: application régulière de lubrifiants et de larmes artificielles; l'achalasie: traitement chirurgical exclusivement (cardiomyotomie de Heller avec geste antireflux) avec un traitement antireflux en post opératoire immédiat afin d’éviter les complications bronchopulmonaires; l'insuffisance surrénale: apport en glucocorticoïdes avec surveillance stricte en milieu hospitalier au début sans omettre le traitement adjuvant (potassium, calcium, vitamine D 3) et un régime hyposodé hypoglycémique. Ce type de pathologie nécessite une surveillance et un suivi multidisciplinaire: ophtalmologique, pédiatrique, neurologique et une éducation du patient sur son hygiène de vie quelque soit son âge.

## Conclusion

Le syndrome d'Allgrove ou triple A syndrome est un trouble pédiatrique rare, associant alacrymie et achalasie qui sont constants et précoces, et une insuffisance surrénale moins constante. Ces troubles sont à l'origine d'une altération de la qualité de vie des patients imposant une prise en charge multidisciplinaire et surtout un conseil génétique dans la fratrie.
